# Tailor-Made Modification of Commercial Ceramic Membranes for Environmental and Energy-Oriented Gas Separation Applications

**DOI:** 10.3390/membranes12030307

**Published:** 2022-03-09

**Authors:** Triantafyllia K. Grekou, Dimitris E. Koutsonikolas, George Karagiannakis, Eustathios S. Kikkinides

**Affiliations:** 1 Department of Chemical Engineering, Aristotle University of Thessaloniki (AUTH), 54124 Thessaloniki, Greece; tkgrekou@certh.gr; 2 Chemical Process & Energy Resources Institute (CPERI), Centre for Research & Technology Hellas (CERTH), 6th km Charilaou-Thermi, Thermi, 57001 Thessaloniki, Greece; dkoutson@certh.gr (D.E.K.); gkarag@certh.gr (G.K.)

**Keywords:** inorganic membranes, silica membranes, gas mixture separation, CVD modification

## Abstract

Ceramic membranes have been considered as potential candidates for several gas separation processes of industrial interest, due to their increased thermal and chemical stability compared to polymeric ones. In the present study, commercial Hybrid Silica (HybSi^®^) membranes have been evaluated and modified accordingly, to enhance their gas separation performance for targeted applications, including CO_2_ removal from flue gas and H_2_ recovery from hydrogen-containing natural gas streams. The developed membranes have been characterized before and after modification by relative permeability, single gas permeation, and equimolar separation tests of the respective gas mixtures. The modification procedures, involving in situ chemical vapor deposition and superficial functionalization, aim for precise control of the membranes’ pore size and surface chemistry. High performance membranes have been successfully developed, presenting an increase in H_2_/CH_4_ permselectivity from 12.8 to 45.6 at 250 °C. Ultimately, the modified HybSi^®^ membrane exhibits a promising separation performance at 250 °C, and 5 bar feed pressure, obtaining above 92% H_2_ purity in the product stream combined with a notable H_2_ recovery of 65%, which can be further improved if a vacuum is applied on the permeate side, leading to 94.3% H_2_ purity and 69% H_2_ recovery.

## 1. Introduction

The intense concerns about global warming and climate change were stressed again during the recent COP26 conference in Glasgow. Taking into account that around two-thirds of greenhouse gas (GHG) emissions globally come from energy production and consumption activities [1], signifies the need for taking action on energy decarbonization. Within this scope, renewable and low-carbon technologies should be broadly deployed so that they end up significantly contributing to the global energy demand. However, considering the reality that fossil fuels will remain a major source of energy for at least a few more decades, the development and integration of efficient carbon capture, usage, and storage (CCUS) technologies is also expected to play an important role in the short term.

Although renewable energy sources such as solar and wind will need to be implemented in abundance in order to meet the global energy demands, they are currently covering only a small share, mainly due to limitations regarding the readiness of energy generation, transportation, and storage infrastructures [2]. Additionally, sun and wind do not provide reliable and continuous energy outputs over time, while surplus electricity is often wasted due to limited storage capacities.

In this direction, a promising long-term energy storage solution is to use the excess renewable electricity for hydrogen production, which is an excellent chemical energy carrier due to its high mass energy density (142 MJ∙kg^−1^) [2]. Hydrogen could then be injected and stored in the existing natural gas grid to be consumed by end-users, provided that suitable H_2_ purifying technologies are in place [3]. Therefore, compact, cost-effective, and efficient technologies for H_2_ recovery from CH_4_ streams must be developed.

However, as stated above, renewables are not ready to dominate the energy sector today, although there is an urgent need to reduce CO_2_ emissions. Achieving the goal of not reaching a 2 °C increase [4] does not preclude fossil fuel usage as soon as efficient CCUS technologies are globally implemented. CCUS will allow fossils fuels to become “part of the solution”, and will provide the required time for the development of future alternative approaches [5].

When it comes to CO_2_ capture from flue gas streams, amine absorption is the current state-of-the-art process that can be implemented at an industrial scale [6,7]. However, although absorption is a well-established process for CO_2_ capture, it is linked with several serious limitations, such as the substantial energy requirements and solvent regeneration difficulties, as well as toxicity and environmental pollution concerns from many of the conventional solvents [8]. Therefore, improved post-combustion carbon capture processes are needed.

Membrane-based separations have attracted growing attention due to their inherent assets related to the energy-efficiency, cost-effectiveness, compact design, ease of operation, and scale-up, compared with conventional processes, such as absorption, cryogenic distillation, and pressure swing adsorption (PSA). Membranes for gas separations have been commercialized for several applications such as CO_2_ removal from natural gas and hydrogen purification in refineries [9]. Although most commercial-scale membranes are polymeric, over the last decades, microporous inorganic and hybrid, and organic–inorganic membranes are getting increased attention. The main advantages of inorganic membranes over polymeric ones are their very good chemical and thermal stability, especially under harsh industrial conditions (high temperature and corrosive impurities), their longer lifetime, and their typically high permeability values.

Amorphous silica membranes are the most-studied nanoporous ceramic membranes due to their ease of production, either by sol–gel synthesis or chemical vapor deposition/infiltration (CVD/CVI), and their high reported performance in gas separation applications. The main limitation of pure silica membranes is their poor stability in feeds containing water vapor. Researchers have managed to overcome this problem by blending organic, hydrocarbon molecules in between silica’s inorganic structure and creating hybrid membranes, which were proven to be suitable for the dehydration of organic solvents by pervaporation [10]. Additional research efforts have focused on tuning silica’s pore structure, or introducing chemical functionalities, based on the targeted gas separation. Some interesting studies using silica membranes and functionalized silica membranes have been reported both for CO_2_/N_2_ [11,12,13,14,15,16,17,18] and H_2_/CH_4_ [19,20,21,22] separations. Although some results are very promising, it is generally accepted that it is extremely difficult to systematically reproduce these nanoporous structures with the same surface characteristics in order to obtain a similar separation performance. Thus, the main challenge when commercializing inorganic microporous membranes is the reproducible production of defect-free large area membranes and modules [23].

Over the last two decades, silica and hybrid silica [10,18] membranes have been commercialized for small scale pervaporation applications. Although HybSi^®^ membranes are among the ones that have been on the market for pervaporation and specifically for solvent dehydration applications, their characteristics render them as potential candidates for various gas separation applications. The scope of the present work is to investigate the separation performance of hybrid silica membranes for environmentally important gas separations, starting from a well-defined, already commercial product. Additionally, these state-of-the-art membranes are being used as a reference point for further development using post-treatment methods, aiming to produce selective membranes for CO_2_/N_2_ and H_2_/CH_4_ separation.

In this direction, we first report some details for the materials we employed, and we extensively describe the experimental procedures we followed for the evaluation and modification of the commercial membranes. Accordingly, we present the experimental test results along with a detailed discussion in comparison with the literature data. Finally, we conclude by pointing out the most important outcomes and suggesting routes for further investigation.

## 2. Materials and Methods

### 2.1. Materials

To the best of our knowledge, there are no commercially available silica membranes tailored for gas separation applications. However, Pervatech BV (Rijssen, The Netherlands) has commercialized hybrid silica membranes (HybSi^®^) for pervaporation applications, which, according to the manufacturer, are considered to have a low defects density and a narrow pore size distribution with pores between 0.3 and 0.5 nm [10], also making them potential candidates for gas separation applications. For this reason, single-channel, tubular, hybrid silica HybSi^®^ with an effective area of 0.005 m^2^ and dimensions of 250 × 10 × 7 mm (length × OD × ID) were obtained from Pervatech BV in order to assess their gas separation potential, either in their original form or after applying a CVD modification step. The membranes have an asymmetric structure, consisting of a macroporous α-Al_2_O_3_ support (providing mechanical stability to the system), one or more inner γ-Al_2_O_3_ mesoporous layers (for gradual pore size reduction) and a final inner organic–inorganic hybrid silica (HybSi^®^) top-layer with hydrophilic surface properties (responsible for membranes’ separation performance). For the chemical vapor deposition methods tetraethoxysilane (TEOS; Alfa Aesar (Ward Hill, MA, USA), 98%) and 3-(triethoxysisyl)-propylamine (APTES; Merk (Darmstadt, Germany), ≥98%) were used as silica and amino-silica precursors, respectively. Finally, H_2_, N_2_, CO_2_, CH_4_, SF_6_, and O_2_ gases with a purity higher than 99.9% were used for the membrane characterization and evaluation.

### 2.2. Methods

The commercial (unmodified) membranes were initially characterized by single gas permeation tests followed by binary CO_2_/N_2_ and H_2_/CH_4_ separation measurements at various conditions. The membranes were then modified according to a low-temperature CVI method, which has been previously described in detail [24,25]. Based on the desired application, the reactant stream used for modification included either TEOS, APTES, and O_2,_ or just TEOS and O_2_. After modification, the membranes were characterized again with both single gas permeation and mixture gas separation tests.

#### 2.2.1. Experimental Apparatus

**Figure 1:**
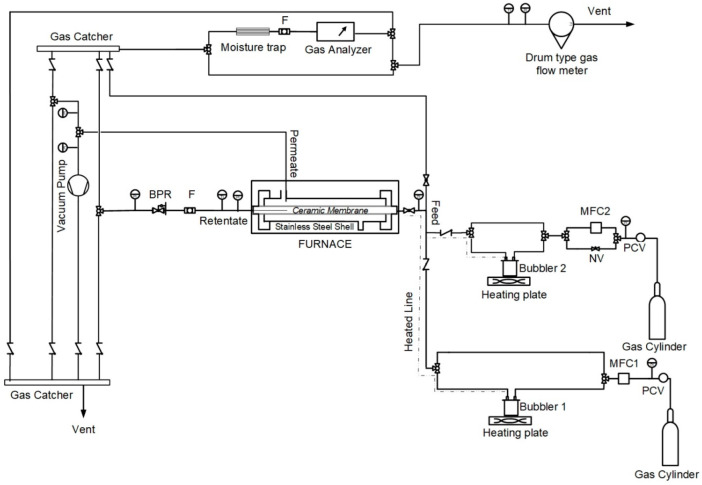
Process and Instrumentation Diagram (P&ID) of the experimental unit.

All of the experiments were held on a custom-made multifunctional rig designed to allow single gas permeation, gas separation, and CVD modification experiments, as described in a previous work [26]. Figure 1 shows the updated Process and Instrumentation Diagram (P&ID), as formed for the needs of the present work. Specifically, the experimental rig consisted of a membrane module connected to a feed, a retentate, and a permeate section. Membranes were securely mounted on a stainless-steel shell and sealed by Kalrez^®^ o-rings (Du Pont, Newark, DE, USA). The shell was placed in the center of a furnace consisting of three independent temperature-controlled zones, providing a uniform temperature profile. On the feed section, two independent lines provided constant feed flow through mass flow controllers (EL-FLOW Prestige, Bronkhorst, Ruurlo, Netherlands) at pressures of up to 10 bar. For both lines, gas could either pass through the silane-consisting bubbler (in case of CVD modification) or bypass it to be sent directly to the membrane. On the retentate side, a back pressure regulator could adjust the desired feed pressure, while on the permeate side there was a possibility to apply a vacuum by a rotatory vacuum pump (ULVAC GLD-040, ULVAC KIKO, Saito, Japan). The feed, retentate, and permeate flows could all be analyzed by an online gas analyzer (Gasboard-3200, from Hubei Cubic-Ruiyi Instrument Co., Ltd., Wuhan, China) and could successively be accurately measured by a drum type gas flow meter (TG1/5, Ritter, Bochum, Germany). The rig was also equipped with the necessary stainless steel tubes, valves, and fittings, as well as with suitable instrumentation for continuous process monitoring and control.

#### 2.2.2. Membrane Performance Evaluation

A HybSi^®^ membrane was first dried at 200 °C under a nitrogen flow and then characterized by single gas permeation tests of various gases (H_2_, CO_2_, N_2_, CH_4_, and SF_6_) at temperatures between 25–250 °C. Each gas was introduced to the inner side of the membrane at a standard pressure of 3 bar, while the permeate side was at ambient pressure. The permeating flow rate was continuously measured by the gas flow meter and, after reaching a steady state, a mean value was used to calculate each gas permeance (mol m^−2^ s^−1^ Pa^−1^), according to Equation (1):

(1) $$\pi_{i}=\frac{F_{i}}{A\cdot \Delta P_{i}}$$

where *F_i_* is the permeate molar flow (mol s^−^^1^) and is calculated from the equation of state of ideal gases using the experimentally found volume flow rate of *i* species. *A* (m^2^) is the membrane area and Δ*P_i_* (Pa) is the partial pressure difference of component *i* through the membrane. The ideal selectivity (permselectivity) of various gas pairs was then calculated as the ratio of the respective permeance values, as shown in Equation (2).

(2) PSij=πiπj

During the gas separation tests, the membrane was fed with an equimolar binary mixture, while the retentate and permeate streams were sequentially measured by the gas analyzer the and gas flow meter. The parameters used to describe the membrane’s separation performance during those tests were the process stage-cut % calculated by Equation (3), the membrane’s separation factor calculated by Equation (4), the product’s recovery % calculated by Equation (5), and the product’s purity %, which was directly measured and is defined as in Equation (6).

(3) $$SC\left(\%\right)=\frac{Total\;Permeate\;flow\;}{Tatal\;Feed\;flow}\times 100$$

(4) βij=(xixj)p(xixj)f

(5) $$R_{i\;}\left(\%\right)=\frac{F_{p,i}}{F_{f,i}}\times 100$$

(6) $$P_{i\;}\left(\%\right)=\frac{F_{p,i}}{\left(F_{p,i}+F_{p,j}\right)}\times 100$$

where *x*_i_ is the mol fraction of gas species, *i*.

CO_2_/N_2_ (50/50) separation tests were performed over a temperature range from 25 °C to 100 °C. The membrane performance was investigated for several feed flow values, through different stage-cut conditions and either by applying vacuum on the permeate side and keeping atmospheric feed pressure or by setting a fixed feed pressure of 6 bar while the permeate pressure was ambient. On the other hand, H_2_/CH_4_ (50/50) separation was performed at 250 °C and at a standard feed pressure of 5 bar, as many European countries use up to 5 bar on their medium-pressure gas distribution network pipelines [27]. The permeate pressure was either atmospheric or maintained under a vacuum.

#### 2.2.3. Membrane Modification by Chemical Vapor Deposition (CVD)

In order to enhance CO_2_ selectivity, the first membrane (M-A) was exposed to a mixture of TEOS, APTES, and O_2_ at 200 °C and 2 bar. A flow of 15 L/h (STP) N_2_ was used as a carrier gas for TEOS vapors obtained from bubbler 1 (50 °C). Additionally, 19 L/h (STP) O_2_ passed through bubbler 2 (23.4 °C) to carry APTES vapors and both flows were preheated at 80 °C by ribbon heaters to prevent the condensation of the reactants. The CVD treatment was carried out in two stages (80 min and a total of 180 min) to study how modification time affects membrane’s separation performance. From now on, the modified M-A membrane will be referred to as M-A80 and M-A180 according to each modification stage.

The second membrane (M-B) was modified in an effort to increase H_2_ permeation. A flow of 60 L/h (STP) O_2_ carrying TEOS vapors obtained from bubbler 1 (50 °C) was introduced into the membrane at 250 °C and 2 bar. The feed mixture was preheated at 80 °C and the CVD procedure lasted 180 min, resulting in the modified membrane M-B180.

At this point, it must be noted that the CVD modification protocol and the selection of the employed process parameters were based on previous experience acquired via relevant past studies by our group [24,25,26] and from some initial exploratory tests to identify their effect on membrane performance.

#### 2.2.4. Characterization of Modified Membranes by Scanning Electron Microscopy (SEM)

Layer thickness determination and surface observation of the modified membranes was carried out by high resolution scanning electron microscopy (SEM), JEOL JSM IT-500. The modified membranes, M-A180 and M-B180, after single gas and binary mixture evaluation, were then broken into pieces, and their top surface and cross-section were analyzed, without any pre-treatment (grinding or polishing), so as to prevent surface deterioration.

## 3. Results

### 3.1. HybSi^®^Commercial Membrane Characterization by Single Gas Permeation Tests

**Figure 2:**
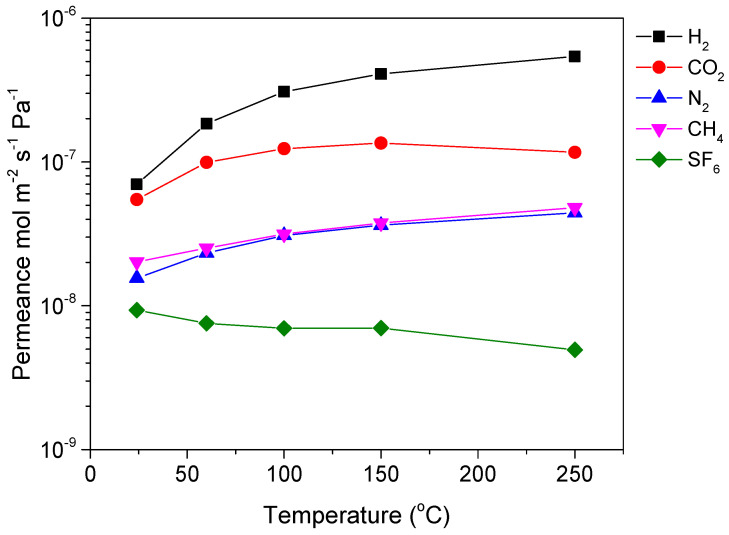
Single gas permeance of various gases as a function of temperature for commercial Hybrid silica (HybSi^®^) membrane M-A.

During the initial evaluation of the commercial HybSi^®^ membrane, nitrogen permeance was found to be stable with pressure, indicating the absence of large defects and macropores, where viscous flow would be the dominant gas transport mechanism [28]. Single gas permeation of various gases (H_2_, CO_2_, N_2_, CH_4_, and SF_6_) was then measured over a wide temperature range from 25 to 250 °C to provide a rough estimation of the membrane’s pore structure and a first indication of its gas separation performance. Figure 2 presents the permeance values for a commercial HybSi^®^ membrane, specifically membrane M-A. It is seen that the permeance of small gases increased with temperature, while that of SF_6_ decreased. This behavior can be explained by a combined activated and Knudsen diffusion gas transport mechanism in the membrane. Specifically, the membranes, as stated earlier, are supposed to have pores in the range of 0.3–0.5 nm, where SF_6_ (dk = 0.55 nm) should not permeate at all. However, the existence of some larger pores/defects, in the mesopore range, is inevitable, thereby inducing leakage diffusion of SF_6_. Typically, activated diffusion prevails in micropores and increases with temperature, while Knudsen diffusion prevails in mesopores/defects and decreases with temperature. Thus, the permeance of small molecules increases with temperature, while that of large molecules decreases with temperature, as it was also observed in a previous study [26]. Table 1 presents the ideal selectivity for various gas pairs along with the theoretical Knudsen values (square root of inverse ratio of molecular weights). H_2_/N_2_, H_2_/CH_4_, and H_2_/SF_6_ permselectivities were considerably higher than the corresponding theoretical Knudsen values, while at the same time, the HybSi^®^ membrane seemed to have a strong affinity for CO_2_, indicated by the significantly high CO_2_/N_2_ and the low H_2_/CO_2_ values. For membrane M-A, CO_2_/N_2_ permselectivity had a maximum value of 4.3 at 60 °C while the maximum H_2_/CH_4_ permselectivity was 11.3 at 250 °C.

### 3.2. CO_2_/N_2_ Separation Performance before and after CVD Modification

HybSi^®^ membranes showed a high potential for the separation of carbon dioxide from nitrogen. CO_2_ (d_k_ = 0.33 nm) and N_2_ (d_k_ = 0.36 nm) have similar kinetic diameters, which make their effective separation through a molecular sieving gas transport mechanism almost impossible. In order to achieve high separation factors along with satisfactory permeance values, it is important to control membrane’s microstructure and surface chemistry [11]. CVD modification using both TEOS and APTES aims to narrow the average pore size and/or the pore size distribution of the membrane while, at the same time, to transfuse the chemical functionality with the amine groups over the membrane’s pore surface. 

**Figure 3:**
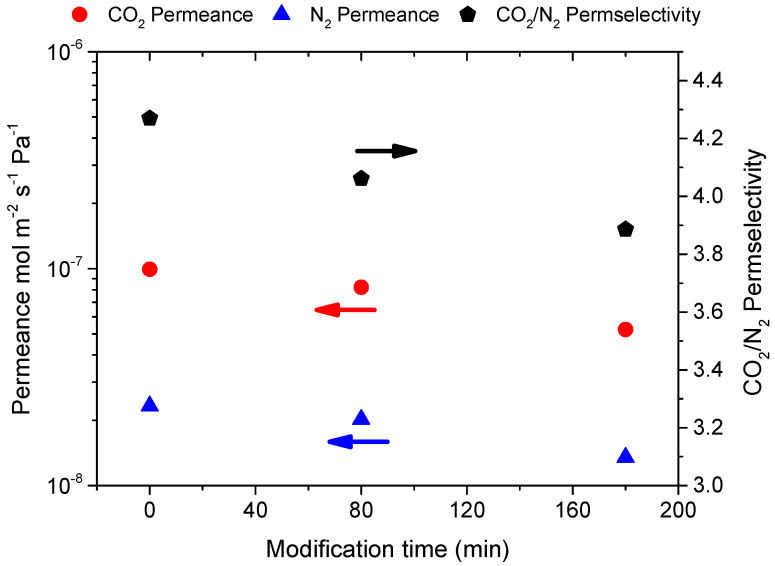
Single gas permeance of CO_2_ and N_2_ measured at 60 °C, P_f_ = 3 bar and their ideal selectivities for membrane M-A as a function of the modification time.

Figure 3 shows the effect of modification time on CO_2_ and N_2_ permeance in M-A membrane. Single gas permeance at T_m_ = 60 °C and P_f_ = 3 bar showed a total reduction of 47% for CO_2_ and 42% for N_2_, resulting in a CO_2_/N_2_ permselectivity reduction from 4.3 to 3.9. The permeance reduction could be attributed to the deposition process, which, although it did not improve the membrane selectivity, it added an extra mass resistance in the system.

**Figure 4:**
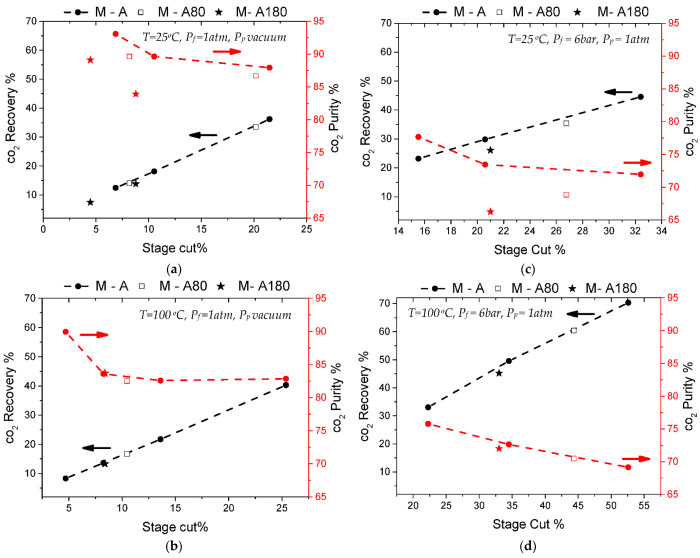
Results of CO_2_/N_2_ (50/50) separation tests at 25 °C (**a**,**c**) and 100 °C (**b**,**d**) either implementing vacuum on the permeate side (**a**,**b**) or 6 bar pressure on the feed side (**c**,**d**). All plots include recovery % and purity % data as a function of stage-cut % for the commercial M-A membrane, compared with results from each stage of its modification (M-A80 and M-A180).

Figure 4 includes the separation test results for the M-A membrane using equimolar CO_2_/N_2_ gas mixtures, before and after CVD modification with TEOS and APTES. Two different separation temperatures were investigated for two pressure-driven conditions. First, the feed pressure was ambient while the permeate side was kept under a vacuum. Then, a second case was investigated setting a standard feed pressure of 6 bar while the permeate pressure was ambient.

The maximum CO_2_ purity on the permeate stream was measured in the commercial membrane M-A, and was as high as 93%, interpreted in a separation factor of 12.8. To obtain that purity level on the product stream, CO_2_ recovery was limited to 12.5%, as the typical purity−recovery tradeoff applied in this case too. These good separation results were obtained at room temperature and under a vacuum on the permeate (Figure 4a). When the feed pressure was applied and the permeate was kept at an atmospheric pressure, the separation performance of membrane M-A was lower, presenting a maximum separation factor of 3.6 at 60 °C, which is in accordance with the single gas permselectivity measurements. These results indicate that the initial inorganic−organic hybrid membrane presented an inherent mild-affinity for CO_2_ that was probably derived from its interaction with the organic groups incorporated in the silica matrix, as proposed before [17,18,29]. These interactions were enhanced at low temperatures and under vacuum conditions at the permeate side. 

During the gas mixture separations, the modified membrane showed significantly decreased separation factors at room temperature (Figure 4a,c), but no considerable differences were observed at a high temperature (Figure 4b,d). This behavior indicates first that the modification did not effectively shift the membrane’s pores size distribution to values where a molecular sieving mechanism would apply, and second, that the mild-CO_2_-affinity, which boosted the initial separation (Figure 4a), was most likely lost after CVD modification. In particular, it was possible that the organic groups incorporated in the silica matrix were affected by the high temperature treatment with O_2_ and were either removed or transformed. 

### 3.3. H_2_/CH_4_ Separation Performance before and after CVD Modification

H_2_ (d_k_ = 0.289 nm) is a small molecule compared to CH_4_ (d_k_ = 0.38 nm), so their separation can be more effectively achieved by narrowing the pores of the membrane. H_2_/CH_4_ separation is expected to show the best results at a high temperature, where Knudsen diffusion through larger pores decreases (negative temperature effect in Knudsen diffusion), while activated diffusion through small pores increases (positive temperature effect in molecular sieving). Temperature dependencies for the permeance due to each transport mechanism were previously explained in detail by J. Gilron and A. Soffer [30].

**Figure 5:**
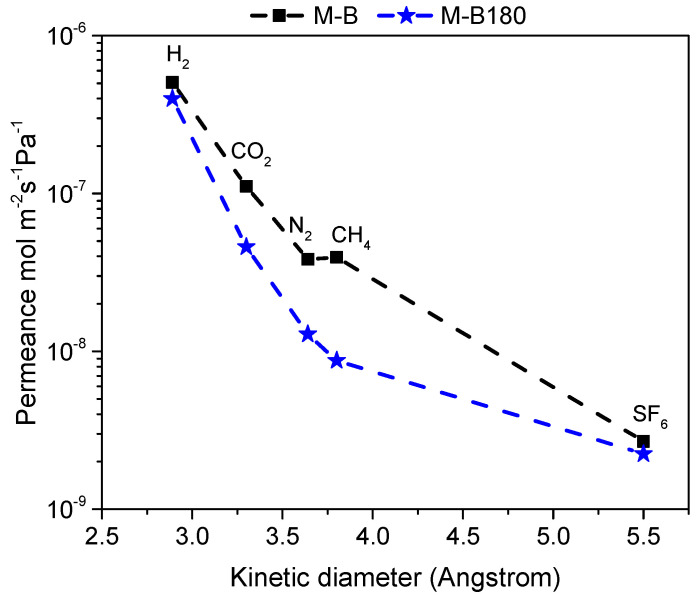
Single gas permeance of various gases measured at 250 °C and P_f_ = 3 bar for commercial HybSi^®^ membrane M-B compared to the modified membrane M-B180 included.

Figure 5 shows single gas permeation at T_m_ = 250 °C as a function of the gases’ kinetic diameter for the HybSi^®^ membrane before (M-B) and after CVD modification (M-B180). Gas permeance on the modified membrane (M-B180) was determined by the kinetic diameter of the molecules, indicating the microporous structure of the membrane. Modification showed almost no influence on hydrogen permeation, but led to a significant reduction of CO_2_, N_2_, and CH_4_ permeance. Therefore, H_2_/CH_4_ permselectivity increased from 12.8 to 45.6, as presented in Table 2. On the contrary, the permeance of SF_6_ was not affected after 3h of modification, suggesting that the few large pores were not sufficiently narrowed to block SF_6_ permeation. This behavior indicated that the pores that were significantly affected were related to the kinetic diameters of the molecules whose permeance was considerably reduced (0.29–0.38 nm). Hence, pores of an effective diameter over 0.55 nm were more difficult to be reduced in size and probably required longer CVD modification or even a slightly higher temperature. At this point, it is important to mention that, based on previous studies from our group [26], H_2_/CH_4_ permselectivity increased with CVD modification, but at the same time, H_2_ permeance decreased. Furthermore, after a certain time period, which typically differs even for membranes from the same production batch, permselectivity reached a plateau and beyond this point, further modification would only decrease membrane’s permeance by producing a thicker top-layer.

**Figure 6:**
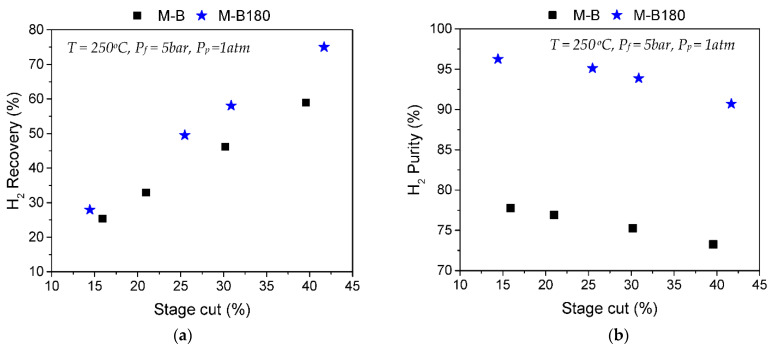
Results of H_2_/CH_4_ (50/50) separation tests at 250 °C, P_f_ = 5 bar and P_p_ ambient. Comparison of (**a**) H_2_ recovery% as a factor of stage cut% and (**b**) H_2_ purity% as a factor of stage cut% between membranes M-B and M-B180.

Figure 6 compares H_2_/CH_4_ separation results at T_m_ = 250 °C between the commercial HybSi^®^ membrane (M-B) and membrane M-B180, derived after CVD modification using TEOS. At a stage cut of 16%, the HybSi^®^ commercial membrane presented a separation factor of 3.7, which could be translated into a limited H_2_ purity of 77.7% (Figure 6b), while its recovery was 25.4% (Figure 6a). After modification, the M-B180 membrane managed to reach a separation factor of 25.9 at a stage cut of 14.4%, meaning that the H_2_ purity increased to 96.3% while 27.9% of the feed H_2_ was recovered on the permeate side.

**Figure 7:**
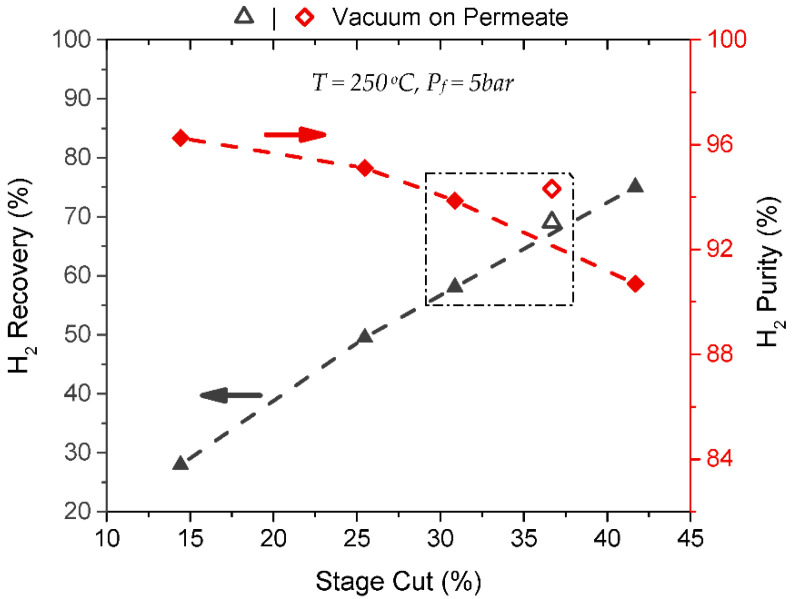
H_2_/CH_4_ (50/50) separation results at 250 °C, P_f_ = 5 bar and P_p_ = 1 atm for modified membrane M-B180 compared to a single separation for which the vacuum was implemented.

The effect of a vacuum was also investigated for H_2_/CH_4_ separation. Figure 7 summarizes the enhanced performance of the modified M-B180 membrane and shows how implementing a vacuum on the permeate influenced the membrane’s separation efficacy. The box-coupled pair of results refers to experiments held at the same temperature, feed pressure, and volumetric feed flow (T_m_ = 250 °C, P_f_ = 5 bar and V_f_ = 66.5L/h at STP). As expected, the implementation of a vacuum shifted the separation to a higher stage cut rate, as the P_f_/P_p_ changed from 4.9 to a very high value of approximately 7.5 × 10^6^. It is well known [9] that the feed-to-permeate pressure ratio is an important process design parameter when separating gas mixtures by a membrane, and a low pressure ratio may even limit membrane’s selectivity. The impementation of a vacuum in combination with a moderate pressure on the gas feed side led to excellent results, providing a high H_2_ recovery of 69% combined with 94.3% H_2_ purity on the product stream.

### 3.4. Scanning Electron Microscopy (SEM) on Modified Membranes M-A180 and M-B180

**Figure 8:**
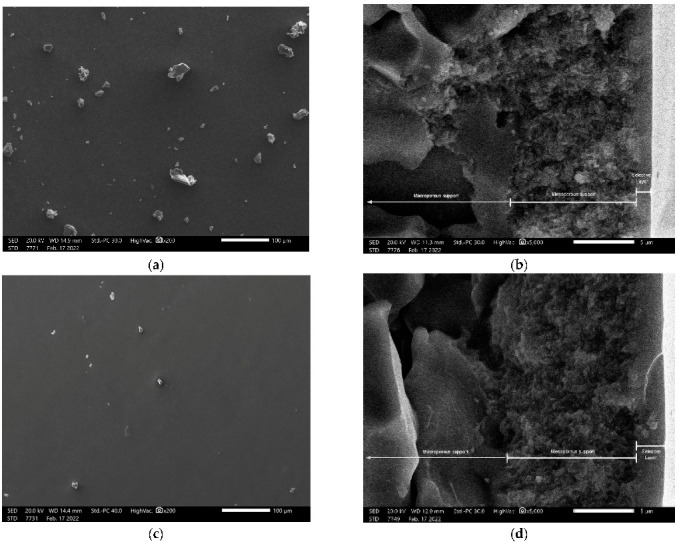
Top-surface (**a**,**c**) and cross-sectional (**b**,**d**) scanning electron microscopy (SEM) images of the modified membrane M-A180 (**a**,**b**) and M-B180 (**c**,**d**).

Scanning electron microscopy (SEM) analysis on membranes M-A180 and M-B180 showed that continuous, crack-free surfaces were formed after both chemical vapor deposition procedures (Figure 8a,c). Cross-sectional images showed the macroporous and mesoporous layers of the supports along with the selective top-layers. M-A180 membrane appeared to have a selective layer thickness of approximately 1 μm (Figure 8b), while the corresponding thickness of the M-B180 membrane was about 2 μm (Figure 8d). It is noted that the superficial debris (Figure 8a,c) as well as the crack-defect on M-B180 selective layer (Figure 8d), are most likely products of each membrane breakage that was necessary for their observation in the microscope.

## 4. Discussion

Although CO_2_/N_2_ is a key-separation to overcome the current environmental crisis, there is limited research on CO_2_-selective microporous silica membranes compared to H_2_-selective ones, for instance. CO_2_ separation from N_2_ is beyond doubt a challenging gas separation. The very similar kinetic diameters and molar masses of the two molecules do not facilitate their separation by molecular sieving techniques or by Knudsen diffusion transport mechanisms. Current efforts are focused on fabricating membranes that could separate CO_2_ by the effect of molecular sieving, along with contributions from its preferable surface diffusion through rigid pores [8,31,32]. Unfortunately, there has been very limited research conducted on chemical vapor deposition either for post-synthesis functionalization or as an initial synthesis route presenting encouraging results. B.A. McCool, W.J. DeSisto [11] investigated the ability of amino functionalization by atomic layer deposition (ALD) to enhance CO_2_ transport in silica membranes. They suggested that high loadings of amino groups, in which interaction with the silica surface was minimized, promoted the highest CO_2_ transport. Suzuki et al. fabricated inorganic−organic hybrid silica membranes, using TEOS and APTES, on porous alumina supports using the CVD method. These membranes exhibited promising CO_2_ permeance of 2.3 × 10^−7^ mol m^−2^ s^−1^ Pa^−1^ coupled with an ideal CO_2_/CH_4_ selectivity of 40 [12].

Inorganic−organic silica membranes have been found to have an inherent mild-affinity for CO_2_ and offer an excellent hydrothermal stability, which is important for many industrially relevant applications that contain relatively small amounts of water [29,33]. These characteristics make them appealing for CO_2_/N_2_ separation, and so this work focused on their evaluation and further modification for that cause. Specifically, HybSi^®^ membranes by Pervatech BV can be described as microporous, high-quality membranes and commercially available with a CO_2_ affinity due to the organic groups incorporated in the silica matrix. The selected membrane presented here exhibited a maximum CO_2_/N_2_ permselectivity of 4.3 at 60 °C coupled with a CO_2_ permeance of ~10^−7^ mol m^−2^ s^−1^ Pa^−1^_._ Gas separation tests with equimolar mixtures showed a high separation factor of 12.8 at ambient temperature and feed pressure by implementing a vacuum on the permeate side. For those separation conditions, 12.5% of CO_2_ was recovered on the permeate side where the CO_2_ purity reached 93%. The HybSi^®^ membrane was consequently modified by CVD, using a mixture of TEOS, APTES, and O_2_ at 200 °C, aiming to narrow the pore openings and transfuse amine-functionality. Contrary to the intended result of this modification strategy, the CO_2_ permeation was hindered rather than boosted. The mixture-gas tests on the modified membrane showed a significant decrease on the separation factor at a low temperature, but no effect at a high temperature. It is believed that the mild-CO_2_-affinity of the initial membrane that led to high separation factors at a low temperature was lost during the CVD treatment. After the modification at high temperature using O_2,_ the membrane’s organic groups, providing CO_2_-selectivity, were most likely removed or transformed. At this point, it is important to add that design parameters like the silane precursor, modification time, temperature, and oxidizing agent certainly play a critical role in the profile of the modified membrane, but a high-quality initial membrane is a prerequisite for a sufficient final separation performance. Thus, it seems that such modifications present a great potential for high quality, defect-free initial membranes, considering that the optimal modification parameters might differ from case to case. 

On the other hand, H_2_ separation by membranes has been broadly investigated for applications related to the syngas ratio adjustment and methane or biogas reforming [34,35], but there are few studies that focus on its separation from hydrogen-enriched natural gas streams. An efficient H_2_ separation from CH_4_ by inorganic membranes needs molecular sieving to be the dominant transport mechanism. Therefore, to achieve both high H_2_ selectivity and permeance requires precise control on the membrane’s morphology: a well-defined, narrow pore size distribution along with a thin active membrane layer. Lee et al. [19] obtained a thin silica layer on a porous alumina support by CVD of TEOS at 600 °C, which presented a H_2_ permeance of ~10^−7^ mol m^−2^ s^−1^ Pa^−1^ with H_2_/CH_4_ selectivity in excess of 1000 at 600 °C. Prabhu and Oyama [22] prepared modified Vycor glass membranes, referred to as Nanosil, by CVD using TEOS at 600 °C with a H_2_/CH_4_ selectivity at up to 23,000–27,000. More studies on silica membranes for hydrogen recovery from hydrogen-enriched natural gas (HENG) can be found in the work of Lu et al. [2], where they discuss and summarize promising membrane technologies for this application.

Commercial HybSi^®^ membranes present H_2_/CH_4_ permselectivities of ~11–13 along with very good H_2_ permeance values of ~5 × 10^−7^ mol m^−2^ s^−1^ Pa^−1^ at 250 °C. Post-synthesis CVD modification by TEOS at a low temperature (250 °C) may sufficiently repair defects in microporous membranes and, as highlighted before [24], offer ease of operation and scale up with the currently available sealing technologies. Such treatment has been successfully implemented on a HybSi^®^ membrane for H_2_/CO_2_ separation by Koutsonikolas et al. [26]. In this study, a H_2_-selective membrane was fabricated out of a commercial HybSi^®^ membrane after CVD modification by TEOS using O_2_ as an oxidizing agent at 250 °C. As a result, H_2_/CH_4_ permselectivity was increased from 12.8 to 45.6, while the equimolar mixture gas tests at 250 °C, P_f_ = 5 bar, and P_p_ ambient led to H_2_ purity above 92%, combined with a notable H_2_ recovery of 65% which was further improved when a vacuum was applied on the permeate side, leading to 94.3% H_2_ purity along with an outstanding H_2_ recovery of 69%.

## 5. Conclusions

Overall, the scope of this work was to evaluate the performance of commercial organic−inorganic hybrid silica (HybSi^®^) membranes for CO_2_/N_2_ and H_2_/CH_4_ separations and to investigate whether their efficiency can be enhanced by applying simple post-synthesis CVD treatments and adjusting operational parameters, mainly the feed to permeate pressure ratio, P_f_/P_p_.

A HybSi^®^ membrane was first evaluated for the separation of binary CO_2_/N_2_ mixture presenting an encouraging separation factor of 12.8 at ambient temperature and feed pressure, by implementing a vacuum on the permeate side. On the permeate side, 12.5% of CO_2_ was recovered and a CO_2_ purity of 93% was achieved. The implemented CVD modification using a mixture of TEOS, APTES, and O_2_ at 200 °C did not meet the expectations of improving the membrane’s separation performance.

A second HybSi^®^ membrane was tested for the separation of H_2_ from CH_4_. CVD modification with TEOS and O_2_ at 250 °C led to a significant increase in H_2_/CH_4_ permselectivity from 12.8 to 45.6. Finally, at 250 °C, P_f_ = 5 bar and P_p_ under a vacuum, the modified membrane succeeded to recover 69% of the supplied H_2_ on the permeate stream combined with a H_2_ purity of 94.3%.

In order to further improve the separation performance of HybSi^®^ membranes for gas separation applications by CVD modifications, research efforts should focus on the effect of the design parameters on the membrane’s final characteristics. Additionally, it is important that each membrane is first fully characterized and then modified by a tailor-made CVD treatment, based on its own characteristics and according to the target-separation.

## Figures and Tables

**Table 1:** Permselectivity values of HybSi^®^ membrane M-A for various gas pairs, compared to the theoretical Knudsen values.

Permselectivity					
T_m_ (°C)	H_2_/N_2_	H_2_/CH_4_	H_2_/SF_6_	H_2_/CO_2_	CO_2_/N_2_
Theoretical Knudsen	3.74	2.83	8.54	4.69	0.80
25	4.5	3.5	7.5	1.3	3.5
60	7.9	7.3	24.5	1.9	4.3
100	10.0	9.8	44.3	2.5	4.0
150	11.3	10.9	58.6	3.0	3.7
250	12.2	11.3	109.8	4.6	2.6

**Table 2:** Permselectivity values of the commercial HybSi^®^ membrane M-B and modified membrane M-B180 for various gas pairs. Theoretical Knudsen values are also presented.

Permselectivity					
Membrane at 250 °C	H_2_/N_2_	H_2_/CH_4_	H_2_/SF_6_	H_2_/CO_2_	CO_2_/N_2_
Theoretical Knudsen	3.74	2.83	8.54	4.69	0.80
Commercial HybSi M-B	13.2	12.8	188.5	4.6	2.9
Modified HybSi M-B180	31.0	45.6	177.8	8.7	3.6

## Data Availability

The data that support the findings of this study are available from the corresponding author, upon request.

## References

[1] World Economic Forum (2015). Scaling Technologies to Decarbonize Energy, Int. Secur. Progr. United Kingdom. http://www3.weforum.org/docs/WEF_GAC_Decarbonizing_Energy_White_Paper.pdf.

[2] Lu H.T., Li W., Miandoab E.S., Kanehashi S., Hu G. (2021). The opportunity of membrane technology for hydrogen purification in the power to hydrogen (P2H) roadmap: A review. Front. Chem. Sci. Eng..

[3] von der Grün G.T.M., Hotopp S., Müller-Kirchenbauer J. (2013). Transport and Usage of Hydrogen via Natural Gas Pipeline Systems. Clean Energy Systems in the Subsurface: Production, Storage and Conversion.

[4] Gao Y., Gao X., Zhang X. (2017). The 2 °C Global Temperature Target and the Evolution of the Long-Term Goal of Addressing Climate Change—From the United Nations Framework Convention on Climate Change to the Paris Agreement. Engineering.

[5] Foster S., Elzinga D. (2013). The role of fossil fuels in a sustainable energy system. UN Chron..

[6] Wang Y., Zhao L., Otto A., Robinius M., Stolten D. (2017). A Review of Post-combustion CO_2_ Capture Technologies from Coal-fired Power Plants. Energy Procedia.

[7] Olajire A.A. (2010). CO_2_ capture and separation technologies for end-of-pipe applications—A review. Energy.

[8] Petrovic B., Gorbounov M., Masoudi Soltani S. (2021). Influence of surface modification on selective CO_2_ adsorption: A technical review on mechanisms and methods. Microporous Mesoporous Mater..

[9] Baker R.W. (2012). Membrane Technology and Applications.

[10] Van Veen H.M., Rietkerk M.D.A., Shanahan D.P., Van Tuel M.M.A., Kreiter R., Castricum H.L., Johan E., Vente J.F. (2011). Pushing membrane stability boundaries with HybSi ^®^ pervaporation membranes Methyl terminating group. J. Membr. Sci..

[11] McCool B.A., DeSisto W.J. (2005). Amino-functionalized silica membranes for enhanced carbon dioxide permeation. Adv. Funct. Mater..

[12] Suzuki S., Messaoud S.B., Takagaki A., Sugawara T., Kikuchi R., Oyama S.T. (2014). Development of inorganic–organic hybrid membranes for carbon dioxide/methane separation. J. Membr. Sci..

[13] Messaoud S.B., Takagaki A., Sugawara T., Kikuchi R., Oyama S.T. (2015). Alkylamine–silica hybrid membranes for carbon dioxide/methane separation. J. Membr. Sci..

[14] Guo M., Kanezashi M., Nagasawa H., Yu L., Ohshita J., Tsuru T. (2020). Amino-decorated organosilica membranes for highly permeable CO_2_ capture. J. Membr. Sci..

[15] Ostwal M., Singh R.P., Dec S.F., Lusk M.T., Way J.D. (2011). 3-Aminopropyltriethoxysilane functionalized inorganic membranes for high temperature CO_2_/N_2_ separation. J. Membr. Sci..

[16] Sakamoto Y., Nagata K., Yogo K., Yamada K. (2007). Preparation and CO_2_ separation properties of amine-modified mesoporous silica membranes. Microporous Mesoporous Mater..

[17] Paradis G.G., Kreiter R., van Tuel M.M.A., Nijmeijer A., Vente J.F. (2012). Amino-functionalized microporous hybrid silica membranes. J. Mater. Chem..

[18] Agirre I., Arias P.L., Castricum H.L., Creatore M., Johan E., Paradis G.G., Ngamou P.H.T., Van Veen H.M., Vente J.F. (2014). Hybrid organosilica membranes and processes: Status and outlook. Sep. Purif. Technol..

[19] Lee D., Zhang L., Oyama S.T., Niu S., Saraf R.F. (2004). Synthesis, characterization, and gas permeation properties of a hydrogen permeable silica membrane supported on porous alumina. J. Membr. Sci..

[20] Moon J.-H., Bae J.-H., Bae Y.-S., Chung J.-T., Lee C.-H. (2008). Hydrogen separation from reforming gas using organic templating silica/alumina composite membrane. J. Membr. Sci..

[21] Gu Y., Ted Oyama S. (2007). Ultrathin, hydrogen-selective silica membranes deposited on alumina-graded structures prepared from size-controlled boehmite sols. J. Membr. Sci..

[22] Prabhu A.K., Oyama S.T. (2000). Highly hydrogen selective ceramic membranes: Application to the transformation of greenhouse gases. J. Membr. Sci..

[23] Xomeritakis G., Tsai C.Y., Jiang Y.B., Brinker C.J. (2009). Tubular ceramic-supported sol–gel silica-based membranes for flue gas carbon dioxide capture and sequestration. J. Membr. Sci..

[24] Koutsonikolas D., Kaldis S., Sakellaropoulos G.P. (2009). A low-temperature CVI method for pore modification of sol–gel silica membranes. J. Membr. Sci..

[25] Koutsonikolas D., Kaldis S., Sakellaropoulos G.P., van Loon M.H., Dirrix R.W.J., Terpstra R.A. (2010). Defects in microporous silica membranes: Analysis and repair. Sep. Purif. Technol..

[26] Koutsonikolas D.E., Pantoleontos G., Karagiannakis G., Konstandopoulos A.G. (2021). Development of H_2_ selective silica membranes: Performance evaluation through single gas permeation and gas separation tests. Sep. Purif. Technol..

[27] CEER (2016). Ceer Benchmarking Report on the Quality of Electricity and Gas Supply-2016: Gas-Technical Operational Quality.

[28] Burggraaf A.J. (1996). Chapter 9 Transport and separation properties of membranes with gases and vapours. Membr. Sci. Technol..

[29] Yu L., Kanezashi M., Nagasawa H., Oshita J., Naka A., Tsuru T. (2017). Pyrimidine-bridged organoalkoxysilane membrane for high-efficiency CO_2_ transport via mild affinity. Sep. Purif. Technol..

[30] Gilron J., Soffer A. (2002). Knudsen diffusion in microporous carbon membranes with molecular sieving character. J. Membr. Sci..

[31] Yu L., Kanezashi M., Nagasawa H., Tsuru T. (2018). Role of Amine Type in CO_2_ Separation Performance within Amine Functionalized Silica/Organosilica Membranes: A Review. Appl. Sci..

[32] Krishna R., Van Baten J.M. (2012). Investigating the relative influences of molecular dimensions and binding energies on diffusivities of guest species inside nanoporous crystalline materials. J. Phys. Chem. C.

[33] Castricum H.L., Qureshi H.F., Nijmeijer A., Winnubst L. (2015). Hybrid silica membranes with enhanced hydrogen and CO_2_ separation properties. J. Membr. Sci..

[34] Maroufmashat A., Fowler M. (2017). Transition of future energy system infrastructure; through power-to-gas pathways. Energies.

[35] Adhikari S., Fernando S. (2006). Hydrogen membrane separation techniques. Ind. Eng. Chem. Res..

